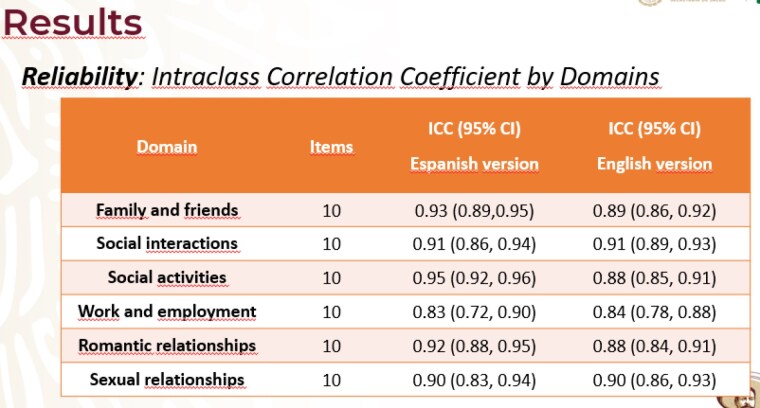# 75 The Life Impact Burn Recovery Evaluation Short Form (LIBRE-SF) Translation, and Validation into Spanish

**DOI:** 10.1093/jbcr/iraf019.075

**Published:** 2025-04-01

**Authors:** Mariana Morales García

**Affiliations:** Instituto Nacional de Rehabilitacion

## Abstract

**Introduction:**

The Life Impact Burn Recovery Evaluation (LIBRE) Computer Adaptive Test (CAT) Profile measures social integration and participation of burn survivors. However, it is not translated into Spanish. A short form version of the LIBRE in English includes 6 domains and 10 items per scale and demonstrates psychometrically sound reliable and valid assessments. We translated from English to Spanish a cultural adaptation of the LIBRE fixed short form (LIBRE-SF).

**Methods:**

Translation methodology followed the ISOQOL and COSMIN guidelines. For test-retest reliability, the questionnaire was administered on 2 occasions at least two weeks apart and included the intraclass correlation coefficients (ICC) Internal consistency reliability with Cronbach Alpha Statistic were applied for results at baseline. Concurrent validity was assessed using the WHODAS 36. Both questionaries were applied in burn patients without open wounds and independent in their daily activities.

**Results:**

The translated LIBRE-SF was applied to 100 patients with burns. We used Cronbach´s Alpha statistics for internal consistency reliability; Intraclass correlation coefficients (ICC) between individual scores obtained in the first and second survey for test-retest reliability (table 1) and Spearman Rho for correlation with WHODAS 2.0 scale.

LIBRE-SF showed a credible level of reliability with Cronbach Alpha Statistic ranged from 0.80 to 0.92, ICC’s ranged between 0.83 and 0.95. Spearman Rho correlations with the WHODAS were significant at the P< 0.05 level for social activities, social interactions and work and employment.

**Conclusions:**

WHODAS assesses more general aspects of function and disability, while LIBRE has more specific focus on social participation after a burn injury. WHODAS is not designed to specifically measure the unique social challenges faced by burn survivors, such as body image or intimate relationships, where the LIBRE is more specific. WHODAS addresses social participation in a broad way and does not consider the psychological and emotional factors that are central to a burn survivor’s experience.

It is likely that some questions were not optimally adapted to the cultural or linguistic context of Spanish speakers and double negative was one of the characteristics of the questions with the lowest internal consistency reliability.Conclusion: The Spanish version is highly reliable, suggesting that patients respond consistently with an ICC between 0.83-0.95, and could be used to assess the emotional needs of burn survivors in Spanish speaking communities, and direct the proper treatment.

**Applicability of Research to Practice:**

A Spanish reliable QoL Scale, would help us to assess and direct treatment.

**Funding for the Study:**

N/A